# Are sex differences in blood cell count and hemoglobin moderated by the 2D:4D ratio? A cross‐sectional study in a Ghanaian population

**DOI:** 10.1002/hsr2.1547

**Published:** 2023-09-04

**Authors:** Moses Banyeh, Thea Kangkpi, Simon B. Bani, Kervin Edinam Zogli, Muniru Mohammed Tanko, Peter Eugene Atuahene, Aisha Yaaba Iddrisu, Christine Ekor, Emmanuel Osei Akoto, Nafiu Amidu

**Affiliations:** ^1^ Department of Biomedical Laboratory Science University for Development Studies Tamale Ghana

**Keywords:** blood cell count, digit ratios, estradiol, Ghana, hemoglobins, testosterone

## Abstract

**Background and Aims:**

There are sex differences in blood cell count and hemoglobin (HGB) in adulthood due to differences in the levels of circulating sex hormones. The second‐to‐fourth digit ratio (2D:4D) is the putative marker of prenatal hormone exposure. The 2D:4D or the right‐left difference (Dr‐l) are sexually dimorphic and are correlates of sex hormones in adulthood. The study sought to determine whether sex differences in adult blood cell count and HGB can be partly explained by the 2D:4D or Dr‐l.

**Methods:**

The study was cross‐sectional between June and December 2021 at the University for Development Studies. The study involved 207 healthy participants (females = 113) aged from 18 to 32 years. The right‐hand (2D:4DR), and the left‐hand (2D:4DL) digit ratio and their difference (Dr‐l) were measured using Computer‐assisted analysis. Blood cell count, HGB, testosterone, and estradiol were measured from venous blood samples using an automated HGB analyzer and ELIZA technique.

**Results:**

The platelet count was inversely related to the 2D:4DR in the total sample with the 2D:4DR accounting for about 0.2% (adj*R* ^2^ = 0.002) of the variability in platelet count. However, there was a sex difference as indicated by the significant interaction between sex and the 2D:4DR on platelet count (*p* = 0.03). The relationship between platelet count and the 2D:4DR was negative in females but positive in males. Also, there was a positive relationship between HGB concentration and the Dr‐l in the total study sample, where the Dr‐l accounted for about 0.6% (adj*R* ^2^ = 0.006) of the variability in HGB concentration. Sex interacted significantly with the Dr‐l on HGB concentration (*p* = 0.01) such that the relationship between HGB and the Dr‐l was positive in females but negative in males.

**Conclusion:**

Prenatal hormone exposure, as indexed by the 2D:4D ratio, may partly account for the observed sex differences in platelet count and HGB levels in adulthood.

## Key points

- There was a significant interaction between sex and the 2D:4DR on platelet count.
- The relationship between platelet count and the 2D:4DR was negative in females but positive in males.
- There was a significant interaction between sex and right‐left difference (Dr‐l) on hemoglobin concentration.
- The relationship between hemoglobin and the Dr‐l was positive in females but negative in males.
- Prenatal hormone exposure may partly account for the observed sex differences in platelet count and hemoglobin levels in adulthood.

## 1 INTRODUCTION

There is a sex difference in the red blood cell (RBC), white blood cell (WBC), and platelet (PLT) counts as well as hemoglobin (HGB) concentration in adulthood. The average male adult tends to have a higher blood cell count and HGB than the average adult female. However, due to population variabilities in genetics and environment, these observations are not universal.^1^, ^2^ The sex differences in blood cell numbers and HGB have been attributed to the effect of sex hormones.^3^, ^4^ Adolescent males, relative to females, experience an increase in HGB mass at puberty as testosterone levels begin to surge. Also, the aged and hypogonadal males (due to Klinefelter's syndrome [KS]) who are administered testosterone may see an improvement in erythrocytosis and HGB mass.^5^, ^6^ Moreover, females with hyperandrogenemia due to congenital adrenal hyperplasia (CAH) may have significantly higher HGB than controls albeit there may be variabilities.^7^

While the mechanism by which Testosterone induces erythropoiesis is poorly understood, it is believed that it stimulates erythropoietin (EPO) production.^3^ Testosterone may also have a direct effect on bone marrow by causing an increase in the production of EPO‐responsive cells. The increase in EPO activity leads to the upregulation of the soluble transferrin receptor (sTfR) activity, which is involved in intracellular iron transportation.^4^ However, there have been inconsistencies in the effect of testosterone on EPO. While males and females differ in their hematocrit and testosterone levels, their EPO and sTfR activities may be comparable.^4^ Previous studies have also shown that despite an improvement in HGB mass and hematocrit following testosterone administration, EPO and sTfR activities may not necessarily increase and may even decrease.^3^, ^6^ This may suggest that testosterone may be acting through other mechanisms other than through EPO and sTfR activities. It may be that testosterone act directly on bone marrow hematopoietic stem cells through insulin‐like growth factor‐1 induction that is mediated by mechanisms via androgen receptors. Testosterone may stimulate the formation of erythroid colony‐forming units, increases intestinal iron absorption and iron incorporation into RBCs as well as HGB synthesis.^4^ Testosterone may also improve hematocrit and HGB by increasing bioavailable iron through the suppressing of hepcidin, a liver‐derived iron‐regulating peptide.^3^ While testosterone may promote erythropoiesis, estrogen tends to inhibit it. The administration of a high dose of estrogen has been shown to cause anemia in both humans and animals.^8^ Estrogen has been demonstrated to play a protective role against erythrocytosis as demonstrated in the lower prevalence of chronic mountain sickness disease or Monge's disease among premenopausal but not menopausal women.^9^ Estrogen protects against erythrocytosis by repressing GATA1, leading to erythroid progenitor cell apoptosis via a direct ligand‐dependent protein–protein interactions.^8^

The Organizational Hypothesis posits that prenatal testosterone (PT) exposure leads to sexual dimorphism in human phenotypic traits including the second‐to‐fourth digit ratio (2D:4D).^10^ The 2D:4D is the putative marker of PT exposure. However, exposure to PT alone does not fully account for the sexual dimorphism in the 2D:4D ratio, but a balanced exposure to both PT and prenatal estrogen (PE) during a narrow window in prenatal development.^11^ Evidence in support of the effect of PT and PE exposure on the 2D:4D ratio has been adduced from persons with CAH, a condition characterized by hyperandrogenemia and also persons with KS or complete androgen insensitivity syndrome (CAIS). Even though there has not been a consensus on them, CAH patients have lower 2D:4D, while KS and CAIS patients have higher 2D:4D ratios compared to controls.^12^, ^13^, ^14^ The 2D:4D ratio and the right‐left difference (Dr‐l) are similar in the pattern given to the observation that a lower 2D:4D of the right hand may be associated with more masculine traits and higher ratios with feminine traits. The 2D:4D or the Dr‐l are negative and positive correlates of PT and PE exposure, respectively, and are lower in females than males on average.^15^ They have also been found to correlate positively with circulating testosterone but negatively with circulating estrogen in adulthood, although this observation has not been universal.^15^, ^16^, ^17^, ^18^ Also, the use of the 2D:4D ratio or the Dr‐l as putative markers of PT and PE exposure has been controversial.^19^ However, an overview and a critical review of the available literature have provided pieces of evidence in support of their validity.^20^, ^21^

Given the above observations, it may be suggested that blood cell count and HGB may be correlated with the 2D:4D or circulating testosterone and estradiol or both. Although previous studies have demonstrated the role of testosterone and estrogen in sex differences in blood cell count,^3^, ^4^ hitherto, no study has examined the role of prenatal hormone exposure (2D:4D) in the sexual dimorphism of blood cell count and HGB concentration in a healthy adult population. The study aimed to determine whether the 2D:4D ratio may partly account for sex differences in blood cell count and HGB concentration in adulthood.

## 2 MATERIALS AND METHODS

### 2.1 Study design and settings

The study was cross‐sectional and was carried out on the Tamale campus of the University for Development Studies between June and December 2021. The University for Development Studies is a multicampus tertiary‐level educational institution in the Northern region of Ghana that offers both under‐ and postgraduate programs in the Medical, Agricultural Sciences, and Education.

### 2.2 Study population

The study involved 207 healthy participants (females = 113 and males = 94) who were between the ages of 18–32 years. The participants were part of a larger study from which some papers have already been published**.** ^22^, ^23^, ^24^ The participants had no known history of fractures that could markedly affect standing height and finger length measurements. They did not also have any known hormonal or hematological abnormalities or chronic diseases. The study was not restricted by one's program of study, cultural, religious, or political affiliation.

### 2.3 Measurements

**Figure 1:**
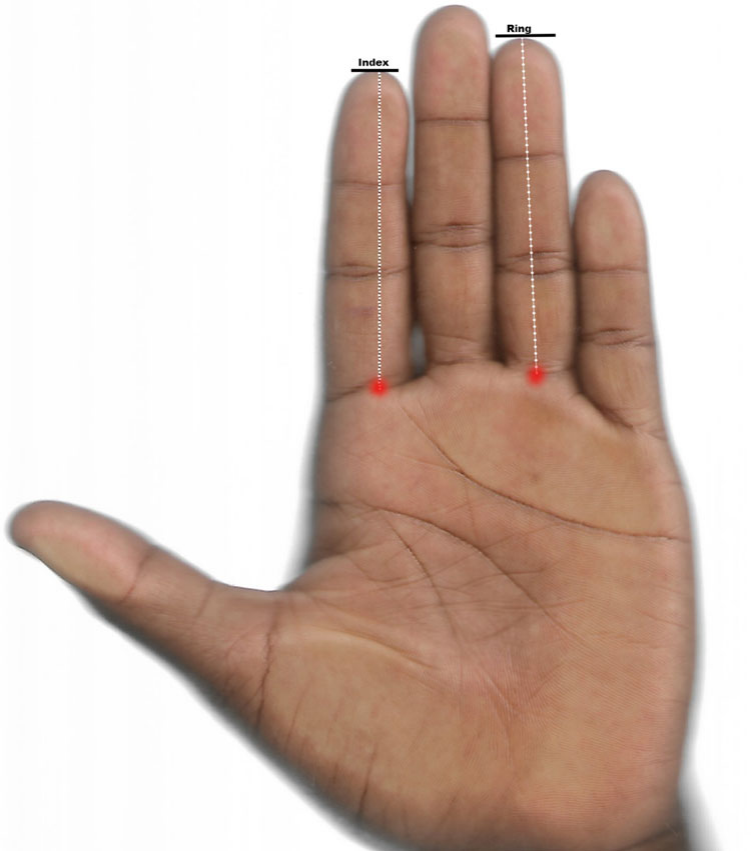
The scanned image of the palmar surface of the hand. The length of the second and fourth fingers was measured from the most proximal crease to the tip of the finger.

The standing height and body weight were measured to the nearest 0.1 cm and 0.1 Kg, respectively, using a stadiometer and body scale. The body mass index (BMI) was then calculated in Kg/m^2^. The second and fourth finger lengths of both hands were measured from hand scans using computer‐assisted analysis.^25^ Each finger length was measured from the most proximal basal crease to the tip of the finger as shown in Figure 1.^26^ Each finger length was measured twice by the same observer and then averaged. The right (2D:4DR) and left (2D:4DL) digit ratios were calculated as the ratio of the second‐to‐fourth digit lengths. The right‐left 2D:4D difference (Dr‐l) was then calculated. The intraclass correlation coefficients (ICC) between the repeated measurements were calculated using the two‐way mixed, single measures with absolute agreement technique. The ICC were 0.966 and 0.950 for the 2D:4DL and 2D:4DR, respectively. Venous blood samples were collected into K_3_EDTA anticoagulated and gel‐separator tubes. The anticoagulated blood samples were analyzed for full blood count within an hour after collection on the Norma Icon‐5 automated hematology analyzer (Elitech Group). The blood in the gel‐separator tube was allowed to clot at 4°C before centrifugation at 1500 rpm for 10 min to obtain serum. The serum samples were aliquoted and stored at −20°C and were never thawed and refrozen until analysis. The serum total testosterone (TT), estradiol, and sex hormone‐binding globulins were measured using ELIZA kits (Monobind Inc.). All the anthropometric measurements and blood sample collection were performed between 8.00‐ and 12.00‐h local time to reduce diurnal variations.

### 2.4 Statistical analysis

All statistical analyses were performed in SPSS (v23) and GraphPad Prism (v8). The data were checked for normality and outliers using the Shapiro–Wilk Test. The data were then summarized as mean ± standard deviation (SD) separately for males and females. The differences in male and female mean values were determined using the student *t*‐test (unpaired, two‐tailed). The Dr‐l was compared to a reference zero value separately for males and females using the one‐sample *t*‐test (one‐tailed). Moderated linear regression models were then formulated with blood cell count and HGB as the dependent variables, while the 2D:4D or circulating hormones were the predictor variables. To reduce multicollinearity, the predictor variables were centered on their means by subtracting the mean value from the variable. Two‐way interaction terms were then created between sex and the centered predictor variables (e.g., Sex*2D:4DR). To reduce confounding, the age at the time of sampling and the BMI were added to each model as covariates. To graphically present the impact of the 2D:4D on sex differences in blood cell count and HGB, the unstandardized predicted values of the dependent variable were plotted on the *y*‐axis against the mean‐centered 2D:4D ratio on the *x*‐axis while the sex variable was made the marking variable. The assumptions of multivariable linear regression were tested using Cooks' distance (Cook's D) for influential multivariable outliers, residual probability–probability plot for multivariable normality, residual scatter plot for homoscedasticity, the variance inflation factor for multicollinearity and the Durbin–Watson test for autocorrelation between the independent variables.^27^ Where there was a violation of an assumption, particularly homoscedasticity, a weight was created from the regression residuals using auxiliary regression analysis. A weighted moderated regression was then performed for the affected model. All statistical analyses were two‐sided at a *p* < 0.05 considered as statistically significant.

## 3 RESULTS

### 3.1 A summary of the study variables

The mean and SDs of the male and female variables are presented in Table 1. Males had significantly higher WBC count and TT than females (*p* < 0.001). However, females had relatively higher RBC count (*p* < 0.001), PLT count (*p* = 0.02), and circulating estradiol (*p* < 0.001) than males. Moreover, females' 2D:4DL was significantly higher than males' (*p* = 0.05).

**Table 1:** The summary of the study variables.

Variable	Female	Male	*t*‐value	*p* Value
BMI (Kg/m^2^)	23.0 ± 3.71	21.6 ± 2.43	3.205	0.001
2D:4DR	0.941 ± 0.035	0.934 ± 0.033	1.53	0.13
2D:4DL	0.943 ± 0.037	0.933 ± 0.034	2.007	0.05
Dr‐l	−0.002 ± 0.026	0.001 ± 0.028	−0.693	0.49
RBC (×10^12^/L)	5.8 ± 1.52	5.1 ± 1.04	3.913	<0.001
WBC (×10^9^/L)	4.6 ± 0.58	5.5 ± 0.45	−12.845	<0.001
PLT (×10^9^/L)	235 ± 68.6	215 ± 52.4	2.265	0.02
HGB (g/L)	12.3 ± 1.12	14.8 ± 1.00	−16.686	<0.001
TT nmol/L	1.6 ± 0.42	21.9 ± 5.60	−33.336	<0.001
E_2_ (pg/mL)	75.0 ± 12.85	32.5 ± 8.04	27.873	<0.001

*Note*: The results are summarized as mean ± SD. The differences in male and female mean values were determined using the student *t*‐test (unpaired, two‐tailed).

Abbreviations: BMI, body mass index; Dr‐l, right‐left difference; HGB, hemoglobin; PLT, platelet; RBC, red blood cell; TT, total testosterone; WBC, white blood cell.

### 3.2 Correlation between 2D:4D and other variables

The Pearson correlation coefficients between the 2D:4D and other variables such as blood cells and circulating hormones are shown in Table 2. The Dr‐l was inversely correlated with RBC (*r* = −0.222, *p* < 0.05) but positively correlated with HGB (*r* = 0.211, *p* < 0.05) in females.

**Table 2:** Correlation between blood cell count, circulating hormones, and digit ratios.

Variable	2D:4DR	2D:4DL	Dr‐l	RBC	WBC	PLT	HGB	TT	E2
2D:4DR	1	0.733	0.317**	−0.138	−0.144	−0.128	0.068	−0.009	−0.120
2D:4DL	*0.656* **	1	−0.413**	0.028	−0.130	−0.059	−0.086	−0.015	−0.133
Dr‐l	*0.390* **	*−0.438* **	1	−0.222*	−0.011	−0.089	0.211*	0.009	0.025
RBC (×10^12^/L)	*−0.023*	*−0.021*	*−0.001*	1	0.084	0.056	−0.192*	0.028	0.092
WBC (×10^9^/L)	*0.051*	*−0.007*	*0.069*	*0.091*	1	−0.248**	0.444**	0.042	0.040
PLT (×10^9^/L)	*0.126*	*0.089*	*0.041*	*−0.111*	*0.013*	1	−0.190*	0.040	−0.007
HGB (g/dL)	*−0.119*	*0.024*	*−0.170*	*0.147*	*0.491* **	*−0.182*	1	0.013	0.041
TT (nmol/L)	*−0.172*	*−0.083*	*−0.105*	*0.129*	*0.176*	*0.020*	*0.182*	1	−0.129
E2 (pg/mL)	*−0.108*	*−0.187*	*0.099*	*0.037*	*0.176*	*0.045*	*0.029*	*−0.035*	1

*Note*: Pearson correlation coefficients for females and males (italicized). The correlation is significant at *p* values.

Abbreviations: Dr‐l, right‐left difference; HGB, hemoglobin; PLT, platelet; RBC, red blood cell; TT, total testosterone; WBC, white blood cell.

* *p* < 0.05

** *p* < 0.01.

### 3.3 Sex difference in PLT count and HGB concentration

**Figure 2:**
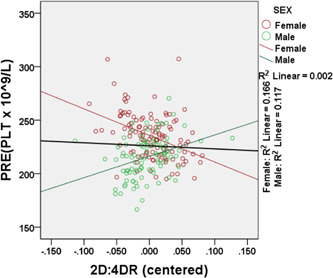
Interaction between sex and the 2D:4DR on adult platelet (PLT) count. The regression‐predicted value of PLT count (PRE) was plotted against the 2D:4DR (centered). The thick black line represents the relationship between PLT and 2D:4DR for the total study sample including males and females. *R* ^2^ = coefficient of determination.

**Figure 3:**
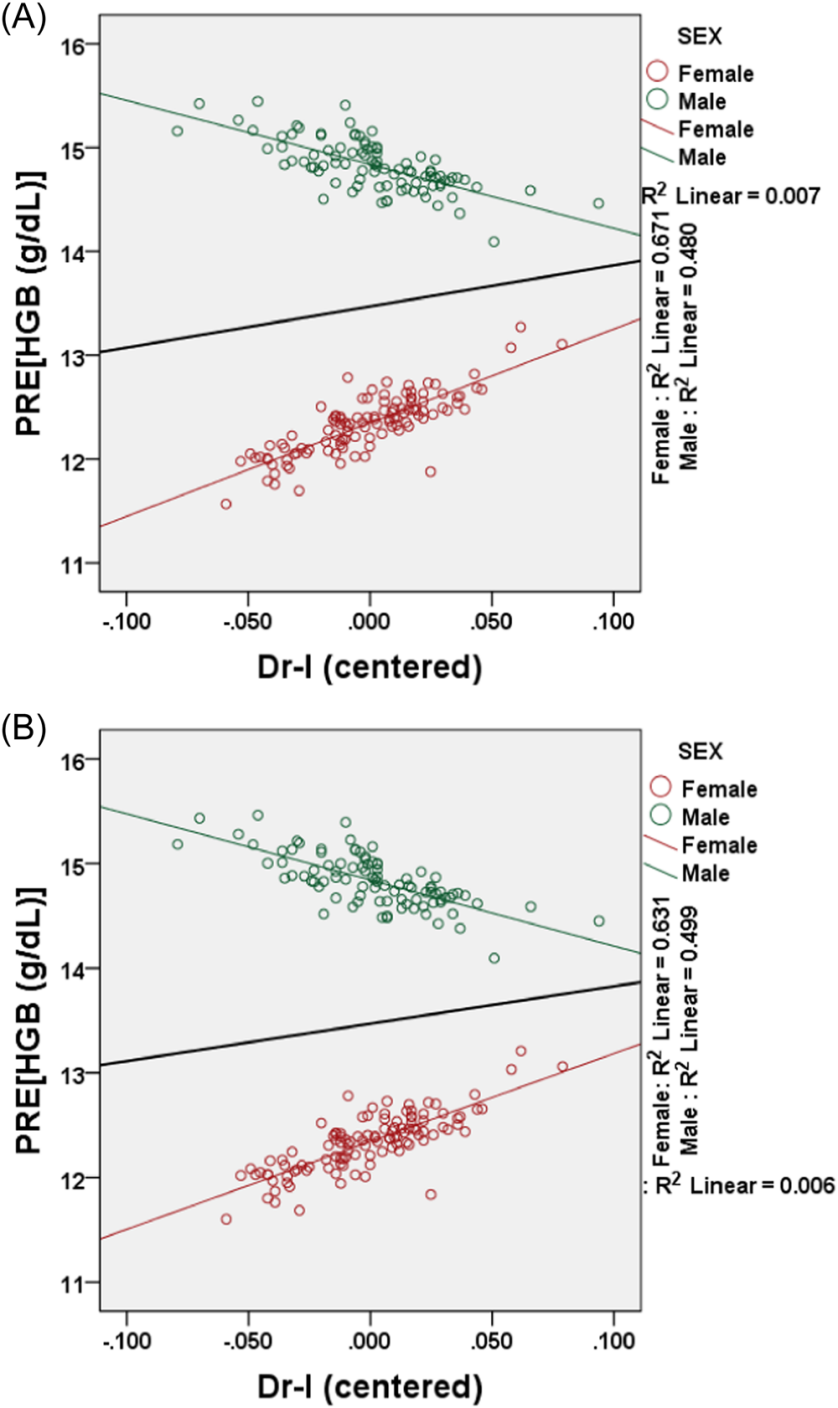
Interactions between sex and right‐left difference (Dr‐l) on hemoglobin (HGB). The regression‐predicted values for HGB (PRE) were plotted on the Dr‐l (centered). The regression analysis was performed unweighted (A) and then weighted (B). The thick black line represents the relationship between HGB and Dr‐l for the total study sample including males and females. *R* ^2^ = coefficient of determination.

The linear regression models with two‐way interaction terms are summarized in Tables 3, 4, 5, 6 and Figures 1 and 2. In general, the PLT count was inversely related to the right‐hand 2D:4D in the total sample and accounted for about 0.2% of the variability in PLT count (Figure 2). However, from Table 5, there was a difference between the sexes as a significant interaction was observed between sex and the 2D:4DR on PLT count (*p* = 0.03). The relationship between PLT count and the 2D:4DR was negative in females but positive in males (Figure 2). The 2D:4DR explained about 17% (adj*R* ^2^ = 0.166) and 12% (adj*R* ^2^ = 0.166) of the variability in PLT count in females and males, respectively. There was a positive relationship between HGB concentration and the Dr‐l in the total study sample (Figure 3). However, there was a significant interaction between sex and the Dr‐l on HGB concentration in both the unweighted (*p* = 0.009) and weighted (*p* = 0.01) regression analysis (Table 6). The relationship was positive in females but negative in males (Figure 3). The assumptions of multivariable regression were tested and interpreted^27^, ^28^ in the Supporting Information. A weighted regression analysis was performed (Figure 3B) to reduce heteroscedasticity (Supporting Information: Figure S2). The assumptions of multivariable regression were tested and interpreted in the Supporting Information and Supporting Information: Figure S1.^27^, ^28^

**Table 3:** Determining the impact of the 2D:4D ratio and circulating hormones on sex differences in RBC count.

LR	RBC (×10^12^/L)	B	95% CI	Upper	*p* Value
			Lower		
1	(Constant)	6.588	4.471	8.704	<0.001
	Age (years)	−0.046	−0.131	0.040	0.30
	BMI (Kg/m^2^)	0.011	−0.047	0.070	0.70
	Sex	−0.669	−1.068	−0.270	0.001
	2D:4DR	−6.036	−13.064	0.991	0.09
	Sex*2D:4DR	5.802	−5.055	16.658	0.29
2	(Constant)	6.576	4.442	8.709	<0.001
	Age (years)	−0.044	−0.131	0.042	0.32
	BMI (Kg/m^2^)	0.010	−0.049	0.069	0.75
	Sex	−0.650	−1.054	−0.246	0.002
	2D:4DL	1.032	−5.777	7.841	0.77
	Sex*2D:4DL	−1.108	−11.754	9.539	0.838
3	(Constant)	6.657	4.569	8.745	<0.001
	Age (years)	−0.044	−0.128	0.040	0.31
	BMI (Kg/m^2^)	0.005	−0.053	0.063	0.86
	Sex	−0.645	−1.037	−0.254	0.001
	Dr‐l	−12.842	−22.156	−3.527	0.007
	Sex*Dr‐l	12.616	−0.833	26.065	0.07
4	(Constant)	7.465	1.676	13.255	0.01
	Age (years)	−0.045	−0.131	0.040	0.30
	BMI (Kg/m^2^)	0.010	−0.048	0.069	0.73
	Sex	−1.795	−7.276	3.687	0.52
	TT (nmol/L)	0.095	−0.497	0.686	0.75
	Sex*TT	−0.070	−0.663	0.523	0.82
5	(Constant)	6.339	4.177	8.501	<0.001
	Age (years)	−0.042	−0.127	0.044	0.34
	BMI (Kg/m^2^)	0.009	−0.050	0.067	0.77
	Sex	−0.370	−1.320	0.580	0.44
	E2	0.010	−0.009	0.030	0.29
	Sex*E2	−0.007	−0.046	0.032	0.74

*Note*: Moderated linear regression analysis with two‐way interaction terms. The predictor variables were centered on their mean to reduce multicollinearity. All analyses were controlled for age at the time of sampling and BMI.

Abbreviations: BMI, body mass index; CI, confidence interval; Dr‐l, right‐left difference; RBC, red blood cell; TT, total testosterone.

**Table 4:** Determining the impact of the 2D:4D ratio and circulating hormones on sex differences in WBC count.

LR	WBC (×10^9^/L)	B	95% CI	Upper	*p* Value
			Lower		
1	(Constant)	4.736	3.900	5.573	<0.001
	Age (years)	0.009	−0.025	0.042	0.62
	BMI (Kg/m^2^)	−0.014	−0.037	0.009	0.22
	Sex	0.906	0.748	1.063	0.001
	2D:4DR	−2.334	−5.111	0.444	0.10
	Sex*2D:4DR	2.918	−1.373	7.210	0.18
2	(Constant)	4.704	3.865	5.544	<0.001
	Age (years)	0.010	−0.024	0.044	0.58
	BMI (Kg/m^2^)	−0.014	−0.037	0.009	0.24
	Sex	0.901	0.742	1.059	<0.001
	2D:4DL	−1.949	−4.627	0.730	0.15
	Sex*2D:4DL	1.690	−2.498	5.877	0.43
3	(Constant)	4.715	3.876	5.555	<0.001
	Age (years)	0.010	−0.024	0.044	0.55
	BMI (Kg/m^2^)	−0.015	−0.039	0.008	0.20
	Sex	0.906	0.749	1.063	<0.001
	Dr‐l	−0.410	−4.155	3.335	0.83
	Sex*Dr‐l	1.618	−3.789	7.026	0.56
4	(Constant)	5.263	2.981	7.544	<0.001
	Age (years)	0.009	−0.024	0.043	0.59
	BMI (Kg/m^2^)	−0.015	−0.038	0.008	0.20
	Sex	0.220	−1.940	2.380	0.84
	TT (nmol/L)	0.058	−0.175	0.291	0.62
	Sex*TT	−0.044	−0.278	0.190	0.71
5	(Constant)	4.644	3.793	5.496	<0.001
	Age (years)	0.012	−0.022	0.046	0.49
	BMI (Kg/m^2^)	−0.016	−0.039	0.008	0.19
	Sex	1.180	0.806	1.555	<0.001
	E2	0.002	−0.006	0.010	0.59
	Sex*E2	0.008	−0.007	0.023	0.31

*Note*: Moderated linear regression analysis with two‐way interaction terms. The predictor variables were centered on their mean to reduce multicollinearity. All analyses were controlled for age at the time of sampling and BMI.

Abbreviations: BMI, body mass index; CI, confidence interval; Dr‐l, right‐left difference; WBC, white blood cell; TT, total testosterone.

**Table 5:** Determining the impact of the 2D:4D ratio and circulating hormones on sex differences in PLT count.

LR	PLT (×10^9^/L)	B	95% CI	Upper	*p* Value
			Lower		
1	(Constant)	262.914	169.111	356.717	<0.001
	Age (years)	−6.371	−10.158	−2.583	0.001
	BMI (Kg/m^2^)	4.914	2.329	7.499	<0.001
	Sex	−3.885	−21.555	13.784	0.67
	2D:4DR	−267.866	−579.343	43.610	0.09
	Sex*2D:4DR	537.317	56.120	1018.514	0.03
2	(Constant)	261.790	167.336	356.243	<0.001
	Age (years)	−6.401	−10.225	−2.577	0.001
	BMI (Kg/m^2^)	4.984	2.377	7.591	<0.001
	Sex	−3.330	−21.214	14.553	0.71
	2D:4DL	−154.288	−455.716	147.139	0.31
	Sex*2D:4DL	388.154	−83.164	859.472	0.11
3	(Constant)	257.652	163.122	352.182	<0.001
	Age (years)	−5.989	−9.796	−2.181	0.002
	BMI (Kg/m^2^)	4.731	2.115	7.348	<0.001
	Sex	−4.770	−22.496	12.956	0.60
	Dr‐l	−182.787	−604.524	238.950	0.39
	Sex*Dr‐l	219.984	−388.938	828.906	0.48
4	(Constant)	311.363	52.882	569.844	0.02
	Age (years)	−6.008	−9.820	−2.196	0.002
	BMI (Kg/m^2^)	4.814	2.200	7.427	<0.001
	Sex	−62.400	−307.101	182.302	0.62
	TT (nmol/L)	5.975	−20.421	32.371	0.66
	Sex*TT	−5.736	−32.222	20.751	0.67
5	(Constant)	259.677	163.122	356.231	<0.001
	Age (years)	−6.033	−9.856	−2.210	0.002
	BMI (Kg/m^2^)	4.827	2.210	7.443	<0.001
	Sex	−4.750	−47.169	37.668	0.83
	E2	−0.156	−1.021	0.709	0.72
	Sex*E2	0.286	−1.458	2.030	0.75

*Note*: Moderated linear regression analysis with two‐way interaction terms. The predictor variables were centered on their mean to reduce multicollinearity. All analyses were controlled for age at the time of sampling and BMI.

Abbreviations: BMI, body mass index; CI, confidence interval; Dr‐l, right‐left difference; PLT, platelet; TT, total testosterone.

**Table 6:** Determining the impact of the 2D:4D ratio and circulating hormones on sex differences in HGB concentration.

LR model	HGB (g/dL)	B	95% CI	Upper	*p* Value
			Lower		
1	(Constant)	11.628	9.942	13.313	<0.001
	Age (years)	0.076	0.008	0.144	0.03
	BMI (Kg/m^2^)	−0.042	−0.089	0.004	0.07
	Sex	2.319	2.002	2.636	<0.001
	2D:4DR	2.361	−3.235	7.957	0.41
	Sex*2D:4DR	−6.790	−15.435	1.855	0.12
2	(Constant)	11.709	10.014	13.404	<0.001
	Age (years)	0.071	0.002	0.139	0.04
	BMI (Kg/m^2^)	−0.040	−0.087	0.007	0.10
	Sex	2.330	2.009	2.651	<0.001
	2D:4DL	−2.271	−7.679	3.137	0.41
	Sex*2D:4DL	1.958	−6.498	10.414	0.65
3A	(Constant)	11.693	10.032	13.353	<0.001
	Age (years)	0.068	0.001	0.135	0.05
	BMI (Kg/m^2^)	−0.037	−0.083	0.009	0.12
	Sex	2.347	2.035	2.658	<0.001
	Dr‐l	8.643	1.234	16.051	0.02
	Sex*Dr‐l	−14.397	−25.093	−3.700	0.009
3B	(Constant)	11.780	10.122	13.438	<0.001
	Age (years)	0.066	−0.001	0.133	0.05
	BMI (Kg/m^2^)	−0.039	−0.085	0.008	0.10
	Sex	2.357	2.047	2.666	<0.001
	Dr‐l	8.012	0.688	15.335	0.03
	Sex*Dr‐l	−13.931	−24.506	−3.356	0.010
4	(Constant)	12.144	7.556	16.731	<0.001
	Age (years)	0.069	0.002	0.137	0.04
	BMI (Kg/m^2^)	−0.041	−0.088	0.005	0.08
	Sex	1.604	−2.739	5.947	0.47
	TT (nmol/L)	0.042	−0.427	0.510	0.86
	Sex*TT	−0.010	−0.480	0.460	0.97
5	(Constant)	11.606	9.883	13.328	<0.001
	Age (years)	0.073	0.005	0.141	0.04
	BMI (Kg/m^2^)	−0.042	−0.088	0.005	0.08
	Sex	2.556	1.799	3.312	<0.001
	E2	0.005	−0.011	0.020	0.54
	Sex*E2	0.001	−0.030	0.032	0.96

*Note*: Moderated linear regression analysis with two‐way interaction terms. The predictor variables were centered on their mean to reduce multicollinearity. All analyses were controlled for age at the time of sampling and BMI.

Abbreviations: BMI, body mass index; CI, confidence interval; Dr‐l, right‐left difference; HGB, hemoglobin; TT, total testosterone.

## 4 DISCUSSION

The study sought to determine whether the 2D:4D or the Dr‐l has an impact on sex differences in blood cell count and HGB in a healthy adult population. It was observed that males had significantly higher HGB but lower PLT than females. The sex difference in the PLT count was moderated by the 2D:4DR, while that of HGB was moderated by the Dr‐l.

It was found that males had significantly higher HGB than females, while females had significantly higher PLT than males. These findings are consistent with previous studies.^1^, ^2^ The sex differences in blood cell count and HGB has been attributed to the effect of sex hormones on hematopoiesis, particularly in males.^6^, ^29^ However, the present study did not observe any significant relationships between blood cell numbers or HGB and adult circulating hormones, but rather with the putative markers of prenatal hormone exposure (2D:4D and Dr‐l). Suggestions of possible correlations between prenatal and adult circulating hormones have been suggested in previous studies.^16^, ^17^, ^26^ If prenatal and adult circulating hormones were correlated, then both the 2D:4D and adult circulating testosterone should have accounted for the sexual dimorphism in the adult PLT and HGB, however, did was not so. The current finding may corroborate the finding of a previous meta‐analysis that showed that prenatal hormone exposure was not correlated with adult circulating hormones.^18^ The possible explanation for prenatal hormones not being correlated with adult circulating hormones may be that Leydig cell populations in the prenatal period degrade by the 24th week of gestation and are then replaced, at puberty and adulthood, by other Leydig cell populations that originate from different stem cells.^30^, ^31^, ^32^

The observation that women with CAH tend to have higher erythropoietic activity than controls may support the suggestion that sexual dimorphism in blood cell count and HGB may occur early in life due to prenatal hormone exposure.^7^ CAH is a hormonal disorder that is characterized by hyperandrogenemia due to 21‐hydroxylase deficiency where the fetus is exposed to excess prenatal androgens.^33^ Although it is not a universal finding, persons with CAH have a lower 2D:4D ratio than controls due to exposure to excess prenatal androgens.^12^, ^13^ It is, however, not clear whether the increased erythropoietic activity is due to prenatal exposure to androgens, the current circulating androgens or both. This will require further studies to clarify the role of prenatal hormone exposure in sexual dimorphism in the hematopoietic pathway.

The current study has some strengths: First, the association between the 2D:4D ratio and sexual dimorphism in blood cell numbers and HGB have not been previously investigated before the current study. Second, the reliability of the linear regression models was assessed by assumption testing and where an assumption was violated, remedial measures were taken. Thirdly, the 2D:4D was measured using computer‐assisted analysis which is more precise than other techniques.^25^ However, the study was limited by the relatively small sample size. Also, genetic and other environmental factors, which may have an impact on hematopoiesis were not assessed. Further studies are recommended.

## 5 CONCLUSION

Sex differences in PLT count and HGB concentration were moderated by the 2D:4D ratio and the Dr‐l, respectively. This may suggest that prenatal hormone exposure may partly account for the sex differences in PLT count and HGB levels in a healthy adult population.

## AUTHOR CONTRIBUTIONS

**Moses Banyeh**: Conceptualization; methodology; project administration; writing—original draft; writing—review and editing. **Thea Kangkpi**: Methodology; writing—review and editing. **Simon B. Bani**: Methodology; writing—review and editing. **Kervin Edinam Zogli**: Methodology; writing—review and editing. **Muniru Mohammed Tanko**: Methodology; writing—review and editing. **Peter Eugene Atuahene**: Data curation; investigation; writing—review and editing. **Aisha Yaaba Iddrisu**: Data curation; investigation; writing—review and editing. **Christine Ekor**: Data curation; investigation; writing—review and editing. **Emmanuel Osei Akoto**: Data curation; investigation; writing—review and editing. **Nafiu Amidu**: Conceptualization; project administration; validation; writing—review and editing.

## CONFLICT OF INTEREST STATEMENT

The authors declare no conflict of interest.

## ETHICS STATEMENT

The study involved human subjects and as such, the guidelines regarding human subject studies as contained in the 1964 Declaration of Helsinki and its later amendments were followed. The study was approved by the institutional review board of the University for Development Studies (N#: UDS/RB/003/21). Written informed consent was obtained from each participant before the study.

## TRANSPARENCY STATEMENT

The lead author Moses Banyeh affirms that this manuscript is an honest, accurate, and transparent account of the study being reported; that no important aspects of the study have been omitted; and that any discrepancies from the study as planned (and, if relevant, registered) have been explained.

## Supporting information

Supporting information.

## Data Availability

The data supporting the results can be obtained from the corresponding author upon reasonable request.
