# Supplementary material for: Are sex differences in blood cell count and hemoglobin moderated by the 2D:4D ratio? A cross‐sectional study in a Ghanaian population
Source: Health Sci Rep. 2023 Sep 4;6(9):e1547. doi: 10.1002/hsr2.1547 (PMC10476464; doi:10.1002/hsr2.1547)
Supplement: Supplementary file 1 — Supporting information. [file HSR2-6-e1547-s001.docx]

**Supplementary material**

**Are sex differences in blood cell count and haemoglobin moderated by the 2D:4D ratio? A cross-sectional study in a Ghanaian population**

**The 2D:4D and blood cell count**

**Testing the assumptions of multivariable linear regression**

The assumptions of multivariable linear regression were tested for the model involving HGB and the 2D:4DR. The following were the outcomes: The Durbin-Watson value was 1.80; the VIF ranged from 1.18-1.99 and the Cook’s D was between 0.00 and 0.19. Also, the assumptions of multivariable linear regression were tested for models involving platelet count and the Dr-1. The following were the outcomes: The Durbin-Watson value was 1.82; the VIF ranged from 1.11-1.90 while the Cook’s D was between 0.00 and 0.17. For model fitness in a multivariable analysis, it is recommended that the Durbin-Watson should be within 1.50-2.50, the VIF should be <10, and the Cook’s D should be <1.00 (Noel, Kemeza, Kiaritha, & Muhwezi; Tranmer & Elliot, 2008).

.

**Supplementary Figure S1.** The assumptions of multivariable normality and homoscedasticity were tested for the model involving haemoglobin and the 2D:4DR. The regression residuals and predicted values were used to test for multivariable normality using the probability-probability plot (top) while homoscedasticity was tested using a scatter plot (bottom). Multivariable normality is indicated by a diagonal line in the P-P plot while homoscedasticity is indicated by a random distribution of data points about the zero line

**Supplementary Figure S2.** The assumptions of multivariable normality and homoscedasticity were tested for the model involving platelet count and the Dr-l. The regression residuals and predicted values were used to test for multivariable normality using the probability-probability plot (top) while homoscedasticity was tested using a scatter plot (bottom). Multivariable normality is indicated by a diagonal line in the P-P plot while homoscedasticity is indicated by a random distribution of data points about the zero line
